# Adipose-Derived Stromal Cells for the Treatment of Knee Osteoarthritis: A Retrospective Study of Clinical Outcomes and Predictive Factors

**DOI:** 10.3390/healthcare14101281

**Published:** 2026-05-08

**Authors:** Mohsen Hussein, Lara Redek Žnidaršič, Lenart Girandon, Nevenka Kregar Velikonja

**Affiliations:** 1 Faculty of Health Sciences, University of Novo Mesto, Na Loko 2, 8000 Novo Mesto, Slovenia; nevenka.kregar-velikonja@uni-nm.si; 2 Artros, Tehnološki Park 19, 1000 Ljubljana, Slovenia; 3 Institute of Cellular Regenerative Medicine, Tehnološki Park 22a, 1000 Ljubljana, Slovenia; 4 Avelana d.o.o., Solska cesta 12, 8222 Otocec, Slovenia; lara.redek@gmail.com; 5 Bio-ReCell d.o.o., Litijska 188, 1261 Ljubljana, Slovenia; lenart.girandon@gmail.com

**Keywords:** adipose-derived stromal cells, knee osteoarthritis, regenerative medicine, treatment efficiency, cell dose, stromal vascular fraction

## Abstract

**Background/Objectives:** Knee osteoarthritis (OA) is a common degenerative joint disease for which conservative treatments often provide limited long-term benefit. Adipose-derived stromal cells delivered as stromal vascular fraction (SVF) represent a minimally invasive orthobiological approach with potential anti-inflammatory and regenerative effects. This study aimed to evaluate the clinical effectiveness and safety of intra-articular autologous SVF therapy and to explore patient- and treatment-related factors influencing outcomes over one year. **Methods:** This single-center retrospective study included 48 patients with knee OA Kellgren–Lawrence (KL) grade II–III treated with a single intra-articular injection of autologous SVF between June 2020 and February 2022. Clinical outcomes were assessed using the Knee Injury and Osteoarthritis Outcome Score (KOOS) at baseline and at 3 and 12 months post-treatment. Associations between clinical outcomes and age, sex, body mass index (BMI), OA grade, and administered cell dose were analyzed. **Results:** Significant improvements were observed in all KOOS domains at 3 months post-treatment (*p* < 0.001). At 12 months, improvements remained significant across domains, although Symptom scores showed slight attenuation. Higher administered cell dose was associated with greater improvement in KOOS Quality of Life (CFU-F indicators, r_s_ = 0.41–0.45, *p* < 0.01) and Sport and Recreation (TNC indicators, r_s_ = 0.36–0.38, *p* < 0.05) at 12 months, while younger age predicted greater QoL improvement and normal BMI was associated with better Symptom outcomes. Radiographic OA severity did not significantly influence treatment response, and sex-related differences were minimal. No serious adverse events were recorded. **Discussion:** SVF therapy was associated with sustained functional improvement and demonstrated a favorable safety profile in patients with moderate knee OA. Although demographic and treatment-related factors showed limited influence, cell dose, BMI, and age may affect selected outcomes. Prospective controlled studies with larger cohorts and longer follow-up are required to optimize patient selection and treatment protocols. **Conclusions:** These findings suggest that autologous SVF therapy may represent a safe and effective complementary treatment option for patients with moderate knee osteoarthritis seeking alternatives to more invasive interventions; however, these results should be confirmed in prospective controlled studies.

## 1. Introduction

Osteoarthritis (OA) is a chronic, inflammatory, and degenerative joint disease and ranks among the most common problems of the elderly population worldwide [1,2]. The World Health Organization (WHO) places it among the top epidemiological diseases globally, with the currently estimated annual incidence of OA at approximately 1040 people per 100,000 inhabitants (p. 13, [2]). In addition to cartilage, it affects various joint and periarticular structures, including soft tissue, bone, synovial capsule and membrane, infrapatellar fat pad, menisci, ligaments, and muscles [1], (p. 14, [2]), [3,4,5]. Consequently, isolated radiographic assessment often does not reflect the actual severity of symptoms [4,6]. Age, obesity, anatomical abnormalities, repetitive identical joint loading, and the presence of metabolic disorders (hypertension, dyslipidemia, diabetes, etc.) increase the risk of OA [2,5], (p. 13, [7]), [8], but the clinical picture can vary greatly despite similar radiographic findings [4,6,9].

Currently available treatment options mainly focus on symptom relief (e.g., analgesics, anti-inflammatory drugs, physiotherapy, and ultimately surgical interventions) rather than on joint restoration [8,10]. Common conservative interventions contribute to limited improvements in pain and function but do not necessarily prevent OA progression and often lack long-term effects, particularly in advanced OA [11,12], (p. 172, [13]). Although exercise-based rehabilitation remains a cornerstone of conservative treatment and can improve pain and function in the short term, its effects are often limited and may not prevent disease progression, particularly in patients with moderate to advanced OA (pp. 19–20, [14]), [15,16]. As a result, there is increasing interest in minimally invasive biological therapies that aim to modulate inflammation and support tissue repair rather than solely alleviate symptoms.

Orthobiological approaches, such as platelet-rich plasma, hyaluronic acid, bone marrow aspirate concentrate, and adipose tissue–derived products, have gained attention as potential disease-modifying strategies for knee OA [12,17]. Among these, therapies based on mesenchymal stromal cells (MSCs) have emerged as particularly promising due to their immunomodulatory, anti-inflammatory, and trophic properties [18,19,20]. MSCs can influence the OA joint environment by inhibiting pro-inflammatory cytokines, promoting macrophage polarization toward a reparative phenotype, reducing cartilage-degrading enzymes, and stimulating endogenous repair mechanisms through paracrine signaling (pp. 177–179, [13]), [20,21,22,23,24,25,26].

Adipose tissue represents an attractive source of therapeutic cells because of its abundance, ease of harvest, and high cellular yield compared with bone marrow [18,27]. Adipose-derived stromal cells can be administered solely or as part of the stromal vascular fraction (SVF), a heterogeneous cell population obtained through mechanical or enzymatic processing of lipoaspirate. SVF contains stromal progenitor cells, endothelial cells, immune cells, and supporting matrix components, which together contribute to its biological activity (p. 2, [18]), [28]. Previous studies have demonstrated that intra-articular administration of adipose-derived stromal cells or SVF can lead to significant short- and mid-term improvements in pain and function in patients with knee OA, with a favorable safety profile [8,20,29].

Despite increasing clinical utilization of mesenchymal stromal cell-based therapies including SVF therapy, several critical aspects of treatment protocols remain insufficiently standardized. At present, there is no consensus regarding optimal cell concentration, application methods, number of treatment sessions, use of scaffolds, use of adjunctive growth factors, or the most appropriate post-treatment rehabilitation strategies, all of which hinder consistent clinical translation and comparison across studies. Additional challenges include variability in cell isolation and production methods, regulatory and ethical considerations [10,30]. Standardized therapeutic approaches should therefore include cell selection criteria, verification of cellular identity and multipotent differentiation potential, clearly defined processing or expansion techniques, dosage specifications, and structured rehabilitation protocols following treatment, which can be achieved by systematic collection and critical evaluation of current clinical evidence and real-world therapeutic experience, enabling progressive refinement of treatment protocols and facilitating the safe and effective translation of regenerative therapies into routine clinical practice [10].

Although several orthobiological approaches have been explored for knee OA, SVF offers distinct advantages that warrant focused investigation: it provides a heterogeneous autologous cell population within a single intraoperative procedure, avoiding the need for culture expansion, repeat harvesting, or exogenous supplementation required by other cell-based therapies [31]. Moreover, unlike platelet-rich plasma or hyaluronic acid, SVF delivers viable stromal progenitor cells capable of sustained paracrine and immunomodulatory activity, while its autologous nature minimizes immunogenic risk [32]. Despite these advantages, recent systematic reviews have identified critical gaps in the SVF evidence base. Ferreira et al. highlighted that most published SVF clinical studies have a low level of evidence and lack standardized reporting of cell product characteristics [33]. Goncharov et al. noted that while safety and short-term efficacy are consistently supported, few studies have systematically examined patient- and treatment-related predictors of clinical outcomes [31]. Most recently, Gao et al. observed that dose–response relationships remain largely uncharacterized, with SVF product data insufficiently reported to allow meaningful comparison across studies [32]. Consequently, limited real-world data are available regarding factors that influence treatment success, particularly in patients with moderate knee OA treated with enzymatically processed SVF.

The present study aimed to evaluate the clinical effectiveness and safety of autologous adipose-derived stromal cell therapy administered as SVF in patients with knee OA Kellgren–Lawrence grade II–III, as well as to investigate the association between clinical outcomes and patient- and treatment-related factors over a one-year follow-up period. The study focused on the application of SVF as a feasible, minimally invasive, single-procedure autologous therapy with a promising biological rationale to provide further clinical evidence to support the continued scientific development and optimization of this treatment approach. Importantly, quality control in this study extended beyond the widely used total nucleated cell count to include the CFU-F assay, providing a more informative assessment of the regenerative potential of the administered product.

## 2. Materials and Methods

### 2.1. Study Design and Ethics

This study was designed as a quantitative retrospective observational clinical study including patients with knee osteoarthritis (OA), Kellgren–Lawrence (KL) grade II–III, who were treated with intra-articular administration of autologous adipose-derived stromal cells delivered as stromal vascular fraction (SVF).

Clinical data were collected from medical records and included pain intensity assessed using the Visual Analog Scale (VAS) and knee function assessed using the Knee Injury and Osteoarthritis Outcome Score (KOOS) questionnaire. Demographic and clinical variables potentially influencing treatment outcomes, including age, sex, body mass index (BMI), OA grade, and administered cell dose, were also analyzed.

Statistical analyses were performed to evaluate clinical changes over time and to assess associations between patient- and treatment-related factors and treatment outcomes. Depending on data distribution, paired or independent samples *t*-tests, Wilcoxon or Mann–Whitney tests, and correlation analyses were applied as appropriate.

The study was conducted in accordance with the Declaration of Helsinki and applicable national ethical regulations. Ethical approval was granted by the National Medical Ethics Committee of the Republic of Slovenia (decision no. 0120-339/2024-2711-7). Permission to access anonymized clinical data was obtained from Artros d.o.o. (Ljubljana, Slovenia), where treatments were performed. All patient data were anonymized prior to analysis in compliance with data protection regulations, and patients retained the right to withdraw consent for use of their data.

### 2.2. Subjects

The study included 48 adult patients with knee OA grade II–III according to the KL classification who received autologous SVF at Artros d.o.o. between June 2020 and February 2022. All patients treated in the defined period were included in the study. Patient inclusion by year was as follows: 14 patients in 2020, 31 in 2021, and 3 in 2022. The majority of patients (n = 40; 83.3%) presented with primary (idiopathic) knee osteoarthritis. The remaining eight patients (16.7%) had secondary OA attributable to prior knee injury or surgery: anterior cruciate ligament (ACL) reconstruction (n = 2), ACL reconstruction combined with subtotal meniscectomy (n = 2), ACL rupture managed non-operatively (n = 1), combined ACL and posterior cruciate ligament (PCL) rupture managed non-operatively (n = 1), total meniscectomy (n = 1), and surgically treated osteochondral lesion (n = 1). No patients had inflammatory arthropathy, crystalline disease, or OA secondary to osteonecrosis or metabolic conditions, as these were exclusion criteria.

Data extracted from clinical records included demographic characteristics (age, sex, BMI), OA grade, baseline pain intensity (VAS), KOOSs across five domains (Pain, Symptoms, Activities of Daily Living, Sport and Recreation, and Quality of Life) recorded before treatment and at 3- and 12-month follow-up visits, as well as radiographic imaging used for OA grading. Analyses were performed on a complete-case basis, including only patients with available data at each follow-up time point. No imputation of missing data was performed.

The study population consisted predominantly of female patients (62.5%), with a mean age of 53.9 ± 10.9 years. OA grade III was present in 66.7% of participants, while 33.3% had grade II disease. More than 90% of patients had elevated BMI (≥25 kg/m^2^) (Table 1).

Inclusion criteria for the treatment were:

- Age over 18 years;
- Diagnosis of knee OA grade II or III confirmed by radiographic imaging according to KL classification;
- Reported knee pain before treatment with a VAS score of at least 1 or more.

Exclusion criteria were:

- Knee OA grade IV according to KL classification;
- Inflammatory arthropathies or systemic connective tissue diseases;
- Alternative musculoskeletal conditions significantly contributing to knee pain or functional impairment, which could affect the ability to isolate symptoms of the affected knee when evaluating treatment effectiveness;
- Prior intra-articular cell or non-cell-based therapy (e.g., hyaluronic acid, platelet-rich plasma, autologous conditioned serum, SVF, MSC) or knee surgery within 12 months before treatment;
- Prior intra-articular corticosteroid injection within 3 months before treatment.

No significant differences were observed between the KL II and KL III groups in terms of sex distribution (χ^2^ = 0.40, *p* = 0.53) or participant age (Welch’s *t*-test: t = 0.33, *p* = 0.74). A higher proportion of patients with elevated BMI was observed in the KL III group compared with the KL II group; however, this difference did not reach statistical significance (Welch’s *t*-test: t = −1.84, *p* = 0.072). Baseline clinical measures showed slightly lower initial VAS and KOOS-5 scores in the KL II group, but these differences were also not statistically significant (VAS: t = −1.60, *p* = 0.12; KOOS-5: t = 0.26, *p* = 0.80) (Table 1).

### 2.3. Cellular Preparation and Evaluation

For the treatment of knee OA, adipose-derived cells were obtained from the patients’ own adipose tissue. Patients meeting inclusion criteria and providing informed consent underwent lipoaspiration under local or general anesthesia. Adipose tissue was harvested primarily from the subcutaneous abdominal region, with the hip region used in one case.

Following two small skin incisions, a suction cannula connected to a vacuum collection system was introduced into the subcutaneous tissue. Up to 500 mL of tumescent solution diluted in physiological saline was infiltrated evenly into both sides of the abdomen using an infiltration cannula (Arthrex^®^, Naples, FL, USA). After an approximately 15 min period to allow tissue infiltration and vasoconstriction, adipose tissue was manually aspirated using a harvesting cannula connected to a VacLok syringe system (Merit Medical, South Jordan, UT, USA) to generate controlled negative pressure.

The harvesting procedure lasted approximately 35 min on average and yielded between 95 and 260 mL of lipoaspirate for further processing. The harvested adipose tissue subsequently underwent enzymatic digestion to disrupt extracellular matrix structures and release the stromal vascular fraction (SVF) and extensive washing steps prior to application.

Perioperative antibiotic prophylaxis (Cefazolin 2 g IV (Pfizer, New York, NY, USA)) was administered approximately 30 min before the lipoaspiration procedure in all patients, consistent with standard surgical practice. No major complications were observed during or after the harvesting procedure, apart from mild transient pain and localized bruising at the donor site.

### 2.4. SVF Preparation

**Figure 1:**
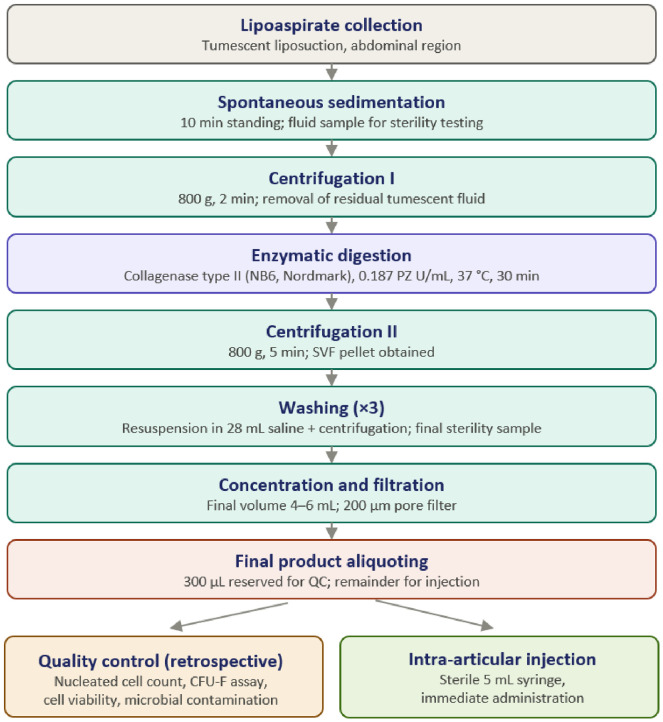
Schematic overview of the intraoperative SVF preparation protocol.

The lipoaspirate was processed intraoperatively using an in-house protocol employing CE-certified medical devices under sterile conditions. Figure 1 illustrates the key steps of the SVF preparation protocol. Initially, the aspirate was allowed to stand for 10 min to permit spontaneous separation of adipose tissue from residual tumescent fluid. A sample of the fluid phase was collected for sterility testing, and the remaining tumescent fluid was discarded.

The adipose fraction was subsequently centrifuged for 2 min at 800× *g* to further remove residual tumescent fluid. The processed adipose tissue (about 80 mL) was then incubated with GMP-grade collagenase type II (NB6, Nordmark, Uetersen, Germany) at a concentration of 0.187 PZ U/mL at 37 °C for 30 min with gentle agitation to facilitate enzymatic digestion.

Following digestion, the suspension was centrifuged at 800× *g* for 5 min to obtain a pellet containing the SVF. The pellet was washed with gentle but firm resuspension in 28 mL of physiological saline solution and centrifuged again. This washing procedure was repeated three times to remove residual enzyme and tissue debris. A final sample of washing solution was collected for sterility control.

After the final centrifugation step, the SVF was concentrated to a final volume of 4–6 mL, filtered through a standard hematological filter (200 µm pore filter) to remove cellular aggregates, and transferred into a sterile 5 mL syringe for intra-articular administration. A 300 µL aliquot of the final product was reserved for laboratory quality control testing, while the remaining suspension was used immediately for injection.

Quality control procedures—performed retrospectively after product administration—included determination of nucleated cell count, colony-forming unit–fibroblast (CFU-F) assay, assessment of microbial contamination, and cell viability testing.

### 2.5. Treatment Procedure

The prepared cellular suspension was administered intra-articularly into the knee joint under sterile conditions. Following standard skin disinfection, the injection was performed using aseptic technique and sterile instruments, after which the puncture site was covered with a sterile dressing.

After the procedure, patients remained under observation with bed rest for approximately two hours and received post-procedural instructions from the orthopedic surgeon.

Patients were instructed to perform a standardized rehabilitation protocol initiated on the day of the procedure. The protocol included knee range-of-motion exercises, isometric strengthening of the quadriceps muscle, and low-intensity aerobic activity. Particular emphasis was placed on early activation of the quadriceps femoris muscle and full active and passive knee flexion and extension. In the presence of pain, participants were instructed to perform movements and exercises up to the threshold of pain onset. Local cryotherapy was recommended after completion of exercises during the first postoperative days. During the first 3–4 months, deep squatting, jumping, and uphill walking, running, or cycling were discouraged, whereas return to sports activities was recommended gradually after 6–9 months, beginning with shorter-duration and lower-load activities followed by progressive increases in loading. Analgesics were prescribed as needed. In addition, all patients were provided with a compression garment to be worn for one month to support donor-site recovery after adipose tissue harvesting.

### 2.6. Clinical and Radiological Evaluation

Clinical outcomes were evaluated using three assessment modalities:

- Pain intensity was measured using the Visual Analog Scale (VAS; range 0–10), recorded prior to treatment.
- Knee function and patient-reported outcomes were assessed using the Knee Injury and Osteoarthritis Outcome Score (KOOS), which includes five domains: Pain, Symptoms, Activities of Daily Living (ADL), Sport and Recreation, and Quality of Life (QoL). KOOS assessments were performed at baseline and repeated at 3 and 12 months following treatment.
- Radiological evaluation was performed prior to treatment using standard knee radiographs and magnetic resonance imaging (MRI) to determine osteoarthritis severity. In one patient, computed tomography (CT) imaging was used instead of MRI due to clinical indications. OA severity was classified according to the Kellgren–Lawrence (KL) grading system, and only patients with grades II and III were included in the analysis.

### 2.7. Statistical Analysis

Statistical analyses were performed using SPSS software (version 25; IBM Corp., Armonk, NY, USA) and Microsoft Excel for Microsoft 365 (version 2602; Microsoft Corporation, Redmond, WA, USA). Data distribution normality was assessed using the Shapiro–Wilk test, with *p* < 0.05 indicating deviation from normal distribution. Descriptive statistics are presented as means and standard deviations, with median and interquartile range (IQR) additionally reported, as several KOOSs significantly deviated from normal distribution. Figures were produced with Claude (https://claude.com) Opus 4.6, an AI tool by Anthropic.

The overall effect of time on KOOSs across the three measurement points (baseline, 3 months, and 12 months) was assessed using the Friedman test, a non-parametric alternative to repeated measures analysis of variance (ANOVA), as Shapiro–Wilk testing indicated significant deviation from normality in 9 of 15 KOOS distributions (5 domains × 3 timepoints; *p* < 0.05), with non-normality most pronounced in the Sport/Recreation and Quality of Life domains due to ceiling effects at later follow-up. A consistent non-parametric approach was therefore adopted across all domains to ensure uniform analytical methodology. The Friedman test was followed by post hoc pairwise comparisons using Wilcoxon signed-rank tests with Bonferroni correction for three pairwise comparisons per domain (adjusted significance threshold: *p* < 0.017). Effect sizes for within-group changes were calculated as paired Cohen’s d (mean within-subject change divided by the standard deviation of individual change scores), with 95% confidence intervals. Conventional thresholds (0.2, 0.5, 0.8) were used to describe effect magnitude.

Differences between independent groups (e.g., sex or BMI categories) were analyzed using Welch’s *t*-test for normally distributed variables or the Mann–Whitney U test for non-normally distributed data. Associations between continuous or ordinal variables, including age, administered cell dose, OA grade, and KOOS outcomes, were assessed using Spearman’s rank correlation coefficient. Statistical significance was set at *p* < 0.05.

Responder analyses were performed to determine the proportion of patients achieving a Minimal Clinically Important Difference (MCID), defined as an improvement of on the KOOS subscale (range 0–100), at each follow-up time point. Domain-specific MCID thresholds were adopted according to Migliorini et al. [31] (Pain ≥ 12; Symptoms ≥ 9; ADL ≥ 10; Sport ≥ 9; QoL ≥ 14). Although these thresholds were derived from total knee arthroplasty populations, they were selected as no validated KOOS MCID values specific to intra-articular cell-based therapies are currently available. Notably, these thresholds are more conservative than the general MCID range of 8–10 points recommended by the KOOS developers [34] and comparable to injection-based OA MCID estimates 8.2–12.5 points [35], thereby providing a stringent benchmark for responder classification. Patients were classified as responders (≥MCID points improvement), non-responders (0–MCID points), or worsened (<0 points) [36].

Multiple linear regression analyses were performed to identify independent predictors of KOOS improvement at 12 months. Models included age, sex, BMI, KL grade, administered cell dose (CFU-F), and baseline KOOS as covariates. Baseline scores were included to control for potential regression to the mean effects. Multicollinearity among predictors was assessed using variance inflation factors (VIF), with values below 5 considered acceptable. Residual normality was verified using the Shapiro–Wilk test. All VIF values were below 5 (range: 1.10–4.23) and residual distributions did not deviate significantly from normality in any model (all Shapiro–Wilk *p* > 0.05). Standardized regression coefficients (β) were calculated to allow comparison of predictor effect sizes. Given the sample size of N = 44 at 12 months and six predictors per model, these analyses should be interpreted as exploratory rather than confirmatory.

## 3. Results

### 3.1. Overall Clinical Outcomes

**Figure 2:**
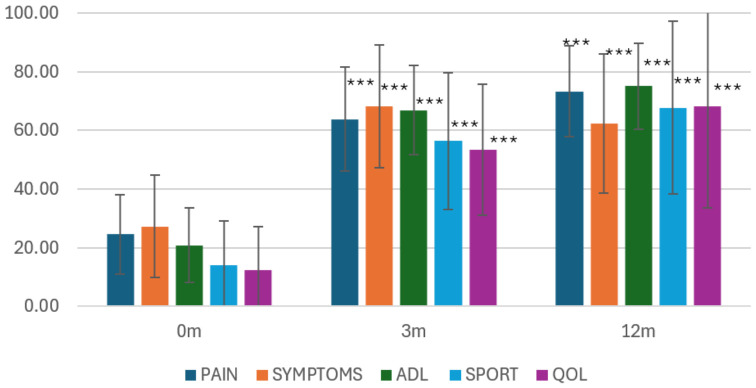
Changes in mean KOOSs over time (M ± SD at 0 months, 3 months, 12 months), presented separately for KOOS-Pain, KOOS-Symptoms, KOOS-Sport, KOOS-ADL, and KOOS-QoL. Legend: *** *p* < 0.001; comparisons to preoperative status (0 m).

Overall clinical outcomes were assessed using KOOSs across all five domains (Table 2, Figure 2). Statistically significant improvements were observed in all domains at 3 months following treatment compared with baseline (all *p* < 0.001), with the largest absolute improvements recorded in the KOOS–Activities of Daily Living (ADL) and KOOS–Pain domains.

At 12 months post-treatment, KOOSs remained significantly improved compared with baseline across all domains (all *p* < 0.001). Comparison between the 3- and 12-month follow-up demonstrated further statistically significant improvements in KOOS–Pain (p_adj = 0.025), KOOS–Sport and Recreation (p_adj = 0.032), and KOOS–Quality of Life (p_adj = 0.024). The KOOS–ADL domain showed a trend toward continued improvement that did not reach statistical significance after correction for multiple comparisons (p_adj = 0.066). The KOOS–Symptoms domain showed a slight non-significant decrease between these time points (p_adj = 0.888).

Overall, the results indicate that the majority of clinical improvements occurred within the first three months after treatment, followed by continued, though smaller, functional gains in most domains up to 12 months, while the Symptoms domain showed a slight decline compared with 3 months.

The Friedman test demonstrated a statistically significant time effect across all five KOOS domains (*p* < 0.001), with large effect sizes indicated by Kendall’s W ranging from 0.647 (Symptoms) to 0.792 (Pain), reflecting substantial and clinically meaningful improvement over time. Paired Cohen’s d values for the baseline-to-12-month interval ranged from 1.25 (Symptoms) to 2.74 (ADL), indicating large to very large treatment effects across all domains. The largest within-period effect sizes were observed during the first three months (d = 3.59–10.18), while effects between 3 and 12 months were small (d = −0.19 to 0.44). After Bonferroni-corrected post hoc testing, statistically significant continued improvement between 3 and 12 months was confirmed for Pain, Sport/Recreation, and Quality of Life, while the ADL improvement trend did not reach corrected significance (p_adj = 0.066). These results confirm that the majority of functional improvements occurred early after treatment, with further modest gains in most domains up to 12 months. Large effect sizes reflect the combination of substantial mean improvement and low variability of within-subject changes, indicating highly consistent treatment response across participants.

#### Responder Analysis

**Figure 3:**
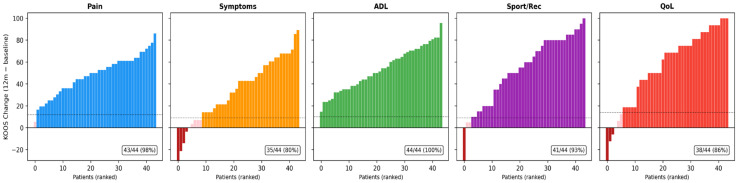
Waterfall plot showing individual patient KOOS improvement at 12 months compared to the status before treatment. Legend: Colored bars indicate patients achieving domain-specific MCID; dark red bars indicate worsening.

Using domain-specific MCID thresholds (Pain ≥ 12; Symptoms ≥ 9; ADL ≥ 10; Sport ≥ 9; QoL ≥ 14), responder rates remained high across domains. At 3 months, virtually all patients achieved clinically meaningful improvement across all KOOS domains (95.8–100%). At 12 months, responder rates remained high, with 100% of patients meeting the MCID threshold for ADL, 97.7% for Pain, 93.2% for Sport/Recreation, and 86.4% for Quality of Life. The Symptoms domain showed the lowest responder rate at 12 months (79.5%), consistent with the observed attenuation in this domain. Notably, no patients experienced worsening in the Pain and ADL domains at 12 months, while 9.1% showed deterioration in Symptoms (Table 3, Figure 3).

### 3.2. Association of OA Grade, BMI, Gender, Age, and Cell Dose with Change of KOOS Domains

#### 3.2.1. OA Grade (KL II vs. KL III)

No statistically significant differences in baseline KOOSs were found between patients with KL grade II and grade III OA (all *p* > 0.05). Furthermore, OA grade was not significantly associated with the magnitude of improvement in any KOOS domain at either 3 or 12 months post-treatment (Table 4).

#### 3.2.2. Body Mass Index

Linear regression analysis showed that BMI was not significantly associated with changes in KOOSs across any domain at any time point, except for the KOOS–Sport and Recreation domain, where BMI was significantly associated with change from baseline to 3 months (B = 0.924, *p* = 0.035) (Table 5).

Additionally, comparison of groups with normal (18.5–24.99 kg/m^2^) and elevated BMI (≥25 kg/m^2^) revealed also a significant difference among groups in symptom perception, as reflected in the KOOS–Symptoms domain, with participants of normal BMI demonstrating significantly better outcomes at 12 months compared with those with elevated BMI (*p* < 0.001).

#### 3.2.3. Age

Age was not significantly correlated with changes in KOOS-Pain, KOOS-Symptoms, KOOS-ADL, or KOOS-Sport and Recreation at any follow-up interval. A statistically significant negative correlation was identified between age and improvement in KOOS–Quality of Life between 3 and 12 months post-treatment (Spearman’s ρ = −0.335, *p* = 0.026), indicating reduced long-term QoL improvement with increasing age (Table 6).

#### 3.2.4. Gender Differences

Baseline KOOS domain analysis revealed a statistically significant difference between male and female patients only in the KOOS–Sport and Recreation domain, with male patients reporting higher activity levels (19.2 ± 13.7) than female patients (10.7 ± 13.2) (*p* = 0.041). No statistically significant sex-related differences were observed in the remaining KOOS domains at baseline.

Following treatment, no statistically significant differences between female and male patients were observed in changes in KOOSs across any domain or follow-up interval (Table 7), indicating comparable treatment-related improvement in both genders.

#### 3.2.5. Cell Dose

High variability of cell product characteristics was demonstrated by post implantation analysis of TNC and CFU in the sample of administered cell preparation. Due to a lack of standardized dosing metrics, the five most common indicators were analyzed (Table 8).

There were no statistically significant differences in cell product characteristics according to gender (*t*-test between groups) or age (Spearman correlation). Although higher BMI was associated with higher numbers of nucleated cells (TNCs) in cell isolate (Spearman’s rho = 0.429, 0 = 0.002) and cell product (Spearman’s rho = 0.368, *p* = 0.010), no statistically significant correlation was found between BMI and no. of CFUs in cell preparations.

Spearman correlation analysis demonstrated no significant association between administered stromal cell dose and early clinical improvement at 3 months across any dose indicator. However, at 12 months, differential dose–response patterns emerged depending on the cell metric used.

TNC-based indicators showed significant positive correlations with improvement in KOOS–Sport and Recreation at 12 months for both TNC/mL of fat tissue (r_s_ = 0.358, *p* = 0.017) and TNC in cell product (r_s_ = 0.377, *p* = 0.012). Additionally, TNC in cell product was significantly correlated with improvement in KOOS–Quality of Life at 12 months (r_s_ = 0.328, *p* = 0.030). No significant associations were observed between TNC indicators and KOOS–Pain, Symptoms, or ADL domains.

CFU-F-based indicators showed significant positive correlations with improvement in KOOS–Quality of Life at 12 months across all three CFU-F metrics (r_s_ = 0.41–0.45, *p* < 0.01), with similar associations for the 3-to-12-month improvement interval. No significant associations were found between CFU-F indicators and KOOS–Pain, Symptoms, Sport, or ADL domains.

No significant dose–response relationships were identified at 3 months for any indicator, and no consistent associations were observed for KOOS–Pain, Symptoms, or ADL at any time point (Table 9).

#### 3.2.6. Multiple Linear Regression Analysis

Multiple linear regression analyses, adjusted for baseline KOOSs, revealed that baseline scores were the strongest independent predictor of improvement in the Pain (β = −0.584, *p* < 0.001), Symptoms (β = −0.549, *p* < 0.001), ADL (β = −0.686, *p* < 0.001), and Sport/Recreation (β = −0.365, *p* = 0.020) domains, indicating that patients with lower initial scores showed greater absolute improvement. BMI was an independent negative predictor of Symptom improvement (β = −0.341, *p* = 0.018), with each unit increase in BMI associated with a 2.1-point reduction in Symptom score change. For Quality of Life, age (β = −0.320, *p* = 0.040) and administered cell dose (β = 0.361, *p* = 0.021) were identified as independent predictors, confirming the bivariate findings and suggesting that younger patients receiving higher cell doses achieved greater QoL improvement. Sex and KL grade did not independently predict improvement in any domain (Table 10).

The KOOS–Sport/Recreation model showed the lowest explanatory power (adj. R^2^ = 0.097) and was the only model with a non-significant overall F-test (*p* = 0.157), indicating that the assessed predictor set did not reliably predict improvement in this domain. This likely reflects the high inter-individual variability in Sport/Recreation outcomes (SD = 32.4; range: −55 to +100 points) and the comparatively weak association between baseline scores and subsequent change in this domain (r_s_ = −0.340).

### 3.3. Safety Outcomes

No serious adverse events were recorded during the follow-up period. Minor local discomfort and transient bruising at the liposuction site were reported but resolved spontaneously without intervention. No cases of infection, prolonged joint swelling, or other treatment-related complications were observed.

## 4. Discussion

This retrospective study evaluated the clinical effectiveness and safety of intra-articular administration of autologous adipose-derived cells delivered as stromal vascular fraction (SVF) in patients with knee osteoarthritis (OA), Kellgren–Lawrence (KL) grade II–III, and investigated patient- and treatment-related factors associated with clinical outcomes over a one-year follow-up period.

The clinical relevance of these findings is further supported by the large effect sizes observed across all KOOS domains (Cohen’s d = 1.25–2.74 at 12 months), which substantially exceed the threshold for large effects (d > 0.8). Furthermore, responder analysis demonstrated that 79.5–100% of patients achieved a Minimal Clinically Important Difference (achieving the domain-specific MCID threshold) at 12 months across all domains, with no patients experiencing worsening in the Pain and ADL domains. These results suggest that SVF therapy provides not only statistically significant but also individually meaningful clinical benefits for the large majority of treated patients. The principal finding of this study is that treatment was associated with statistically significant improvements in pain, function, and quality of life across all KOOS domains at 3 months post-treatment, with improvements largely maintained at 12 months. The most pronounced gains occurred during the first three months, while further, though smaller, improvements were observed in most functional domains between 3 and 12 months. These findings are consistent with previous studies reporting early symptomatic improvement following MSC- or SVF-based intra-articular therapies, likely reflecting rapid immunomodulatory and anti-inflammatory effects within the OA joint environment rather than structural tissue regeneration [8,10,20,23,37].

### 4.1. Influence of Demographic Factors and Cell Product Characteristics

#### 4.1.1. OA Grade

Radiographic OA grade within the moderate disease spectrum (KL II vs. III) did not significantly influence treatment response. This finding supports the view that radiographic assessment of knee OA is not always a reliable prognostic indicator of subjective symptoms or therapeutic response. The discrepancy between radiological findings and clinical presentation likely reflects the multifactorial nature of OA, in which synovial inflammation, periarticular soft tissue changes, cartilage degeneration, and subchondral bone remodeling all contribute to symptom development. Consequently, patients with similar radiographic findings may experience markedly different levels of pain and functional limitation [4,6,9].

The absence of detectable differences between KL II and KL III groups in our cohort may partly be explained by the relatively homogeneous study population, which did not include patients with advanced KL IV disease. Previous studies have reported that clearer outcome differences tend to emerge primarily when end-stage OA is included in the analysis. Conversely, some authors have observed poorer treatment responses in patients with higher OA grades or in older populations [38]; however, because our study population was restricted to moderate OA stages, such differences were not observed. Age-related differences in outcomes were mainly reflected in medium-term quality of life changes rather than in overall functional recovery, supporting previous findings that individual patient characteristics and comorbidities may play a more important role than radiographic severity alone [38]. Overall, these findings suggest that treatment effectiveness in moderate knee OA (KL II–III) appears broadly comparable across these stages, while more pronounced differences in outcomes may become evident only when advanced disease stages are included.

#### 4.1.2. BMI

Our results indicate that elevated BMI primarily affects symptom perception, as reflected in the KOOS–Symptoms domain, with participants of normal BMI (18.5–24.99 kg/m^2^) demonstrating significantly better outcomes at 12 months compared with those with elevated BMI (≥25 kg/m^2^). No significant differences were observed in the remaining KOOS domains. This finding partially aligns with previous reports suggesting that increased body weight adversely influences certain OA-related parameters, particularly pain and inflammatory activity within the joint (p. 2, [18]).

Experimental and clinical studies further suggest that obesity, particularly at BMI values exceeding 30 kg/m^2^, may negatively influence adipose-derived mesenchymal stromal cell (SVF containing AD-MSC) biology by accelerating cellular senescence and reducing regenerative and differentiation capacity, potentially leading to less effective joint tissue recovery (p. 2, [18]), [39]. AD-MSCs obtained from individuals with elevated BMI have been shown to exhibit increased expression of senescence-associated genes (p16, p21, and p53), enhanced secretion of inflammatory mediators such as IL-6 and MCP-1 and reduced proliferative potential [39].

Although BMI does not appear to consistently influence the absolute yield of AD-MSCs, some studies report higher cell harvest efficiency in donors with BMI <25 compared with those in the BMI range of 25–29, while donors with BMI >30 may demonstrate increased adipogenic differentiation potential [40]. In contrast, our study demonstrated a significant positive correlation between BMI and TNC yield (r_s_ = 0.429, *p* = 0.002) but did not find a significant association between BMI and CFU-F yield, suggesting that higher adipose tissue mass increases total nucleated cell recovery without proportionally increasing the stromal progenitor fraction.

Methodological differences in tissue processing, donor characteristics, or sample size limitations may partly account for discrepancies reported across studies [18].

Taken together, these findings suggest that although regenerative cell yield may not be directly impaired, elevated BMI may still negatively influence clinical outcomes through mechanical overload, metabolic factors, and altered cellular function. Therefore, careful monitoring of clinical symptoms, physical function, and metabolic status remains advisable in patients with elevated BMI undergoing AD-MSC therapy, and weight management strategies may contribute to optimizing long-term treatment outcomes.

#### 4.1.3. Age

Our results indicate that age did not significantly influence improvement across most KOOS domains, with a statistically significant negative correlation observed only for quality of life outcomes at 12 months after treatment (Spearman’s ρ = −0.335; *p* = 0.026). This suggests that older participants experienced less pronounced long-term improvement in perceived Quality of Life, whereas improvements in Pain, Symptoms, Sports Function, and Activities of Daily Living were not significantly age dependent. These findings are consistent with reports indicating that the regenerative and chondrogenic potential of adipose-derived mesenchymal stromal cells (AD-MSCs) does not decline substantially with donor age. At the same time, the observed reduction in long-term QoL improvement among older patients may reflect age-related comorbidities, decreased physical adaptability, or broader functional and psychosocial factors influencing perceived recovery [18].

However, other studies have reported that younger donors, particularly those under 30 years of age, may exhibit higher AD-MSC yields and faster cellular proliferation compared with donors older than 50 years, potentially translating into greater clinical improvement in younger patients. For example, cell yields in donors aged 10–29 years have been reported to be approximately twice those observed in individuals aged 50–59 years [40]. Additional studies excluding older patients with advanced OA have also demonstrated greater treatment effectiveness in younger individuals with mild to moderate disease, emphasizing the importance of patient selection and earlier intervention in determining treatment success [38,41].

#### 4.1.4. Cell Dose

Our study examined the relationship between administered cell dose and clinical outcomes using both TNC (total nucleated cell count) and CFU-F (colony-forming unit–fibroblast) as dose indicators. Higher CFU-F counts were significantly associated with greater improvement in KOOS–Quality of Life at 12 months, while TNC counts showed significant positive correlations with improvement in the KOOS–Sport and Recreation domain at 12 months. TNC in the cell product was additionally correlated with QoL improvement. No significant dose–response relationships were observed in Pain, Symptoms, or ADL domains, nor at the 3-month follow-up, regardless of the dose indicator used.

These findings are in line with previous reports indicating that dose-dependent effects of stromal cell therapy may be more evident in longer-term patient-reported outcomes rather than in early symptom relief [29,42]. This observation supports the hypothesis that higher numbers of administered cells may enhance or prolong regenerative and immunomodulatory effects within the joint, although the available literature remains inconsistent regarding optimal dosing strategies [43]. These findings are consistent with the observation by Kolar et al. (2024) that CFU-F counts and their proportion among total nucleated cells (CFU-F/TNC) were positively correlated with improvements in patient-reported outcomes following bone marrow aspirate treatment of osteochondral lesions [44], which indicates similar regenerative efficiency and mechanisms of stem cells from different cell sources.

Meta-analytic data suggest that higher cell doses may confer an initial advantage in pain and functional improvement during the early post-treatment period (3–6 months), with this benefit diminishing over time but still remaining favorable at 12 months [29]. At the same time, some studies indicate that mild transient adverse reactions may occur more frequently with higher cell doses [29], emphasizing the need to balance potential clinical benefits against safety considerations when determining treatment protocols [29,43].

#### 4.1.5. Gender

Our findings indicate that sex did not significantly influence outcomes in most KOOS domains, including Pain, Sport and Recreation, Activities of Daily Living, and Quality of Life. A statistically significant difference was observed only in the KOOS–Symptoms domain at 12 months, with female patients demonstrating greater symptom improvement than male patients. This finding may suggest a degree of sex-related variability in symptom perception or response to treatment; however, its clinical relevance remains uncertain.

Although there are experimental studies reporting that mesenchymal stromal cells derived from male donors may exhibit higher proliferative capacity and increased production of cartilage matrix components, including type II collagen [40,45], several clinical investigations have reported no substantial sex-related differences in treatment outcomes, indicating considerable heterogeneity in the available evidence. Consequently, the isolated difference observed in the present study should be interpreted cautiously and may reflect differences in symptom perception, pain reporting, or other patient-related factors rather than intrinsic biological differences in regenerative response.

Multiple regression analyses identified baseline KOOSs as the strongest predictor of absolute improvement in most domains, a finding that should be interpreted in the context of possible regression to the mean effects inherent in observational studies with baseline measurements. Importantly, after adjusting for baseline scores and other covariates, BMI retained an independent negative association with Symptom improvement, while cell dose and age remained independent predictors of QoL improvement, confirming the bivariate findings reported in the present study.

### 4.2. Clinical Implications

These findings support the potential role of SVF therapy as a complementary option for patients with moderate knee OA who seek to defer or avoid more invasive interventions. The principal clinical significance of our results lies in the durability of treatment effects: functional gains were not only maintained but slightly improved across most KOOS domains between 3 and 12 months, indicating that the therapeutic response extends beyond the immediate post-treatment period. A similar positive clinical effect was reported by Kim et al., 2023 [46], whose meta-analysis of randomized controlled trials demonstrated that both autologous culture-expanded AD-MSCs and SVF preparations produced significant pain improvement at 12 months after intra-articular injection. While patient-related factors appear to have limited influence on most outcomes, optimization of cell dosing, rehabilitation protocols, and patient selection criteria—particularly regarding BMI and age—may further improve results in specific subgroups.

The clinical relevance of the observed improvements is further underscored by the responder analysis. The MCID thresholds applied in this study (Pain ≥ 12; Symptoms ≥ 9; ADL ≥ 10; Sport ≥ 9; QoL ≥ 14) were adopted from Migliorini et al. [31], who derived them from TKA populations. Because our thresholds are at the upper end of the published MCID range, the reported responder rates (79.5–100% at 12 months) represent conservative estimates. These responder rates compare favorably with those reported in other SVF studies. Rogers et al. (2024) reported that 79.3% of patients exceeded the average KOOS MCID at 12 months following GMP-manufactured enzymatic SVF injection in KL grade II–III OA, with domain-specific responder rates of 66–79% [47]. Totlis et al. (2025) observed an 87.0% KOOS MCID responder rate at 12 months in patients receiving mechanical SVF combined with PRP, significantly outperforming hyaluronic acid (57.4%) [48]. Mangiavini et al. (2025) similarly reported that the majority of patients achieved clinically meaningful KOOS improvement after mechanical SVF injection, with NRS pain MCID responder rates of 86.2% at 12 months [49].

The absence of serious adverse events, combined with the self-limiting nature of donor-site reactions reported here and in the existing literature [8,18,28], supports the viability of this approach in routine clinical settings, provided that autologous preparations and appropriate procedural standards are maintained.

SVF therapy, as performed in this study, involves a single-session, same-day surgical and injection procedure and therefore entails costs associated with lipoaspiration, on-site processing, intra-articular injection and subsequent quality control of the administered product. Compared with standard conservative treatments such as physiotherapy, corticosteroids or hyaluronic acid injections, SVF therapy is currently more costly and not reimbursed in most healthcare systems. However, compared with knee replacement surgery, it represents a substantially less invasive and lower-risk option, especially in younger patients trying to postpone knee replacement surgery. If confirmed in controlled trials, its cost-effectiveness relative to repeated conservative treatments or surgical delay strategies would warrant dedicated health-economic analysis.

### 4.3. Limitations

This study has several limitations. Its retrospective observational design limits causal inference and increases susceptibility to selection bias. The relatively small sample size reduces statistical power and may limit the detection of weaker associations. The observed improvements cannot be definitively attributed to SVF therapy, as the absence of a control group precludes ruling out contributions from placebo effects, regression to the mean, or natural disease fluctuation. All multivariate models were therefore adjusted for baseline scores to mitigate this concern.

Although very large effect sizes were observed (particularly in the early post-treatment period, with Cohen’s d values exceeding 3 in several domains), these magnitudes should be interpreted with caution. In within-subject designs, effect sizes are influenced not only by the absolute magnitude of change but also by the variability of individual change scores. In our cohort, relatively low variability in treatment response, combined with substantial baseline impairment and large absolute improvements, contributed to inflated effect size estimates.

Although inclusion of all consecutive eligible patients minimizes selection bias within the study center, the sample may not fully represent the broader knee OA population. Patients treated at Artros d.o.o. self-selected for SVF therapy, suggesting higher socioeconomic capacity, willingness to undergo a minimally invasive procedure, and potentially higher health engagement than the general OA population. Furthermore, the exclusion of patients with recent prior intra-articular treatments or advanced OA (KL IV) increases internal validity but limits generalizability to first-line treatment candidates with moderate disease.

Furthermore, SVF represents a heterogeneous cellular product rather than purified culture-expanded MSCs, limiting direct comparability with other studies. Rehabilitation protocols were not fully standardized, which may have influenced functional outcomes.

The regression model for the Sport/Recreation domain had low explanatory power and a non-significant overall F-test, and therefore the individual predictor estimates from this model should be interpreted with caution. Additionally, the ratio of observations to predictor variables in the multiple regression models (approximately 6.5:1) was below the commonly recommended threshold of 10:1 [50], which may increase the risk of overfitting. The regression results should nonetheless be considered exploratory and require confirmation in larger, adequately powered samples.

Importantly, follow-up was limited to one year, and longer-term treatment durability remains to be established.

Finally, the potential influence of unmeasured confounding factors should be considered when interpreting the results. Variables such as baseline physical activity level, adherence to the rehabilitation protocol, use of concomitant therapies (e.g., analgesics, supplements), psychosocial factors, and patient expectations were not systematically recorded and may have influenced treatment outcomes. Additionally, biological variability in SVF composition beyond the measured parameters (TNC and CFU-F), including differences in cell subpopulations and secretome profiles, may represent another source of unmeasured confounding.

## 5. Conclusions

This study demonstrates that intra-articular administration of autologous adipose-derived cell therapy, delivered as stromal vascular fraction, is associated with significant improvements in pain, function, and quality of life in patients with moderate knee OA (KL grade II–III), with these effects largely maintained over a one-year follow-up period. The clinical relevance of these findings is supported not only by effect size metrics but also by the high proportion of patients achieving MCID thresholds across all KOOS domains, which provides a more patient-centered measure of meaningful improvement. Clinical improvement was most pronounced during the first three months after treatment, followed by stabilization of outcomes in most functional domains throughout the remainder of the observation period.

Patient-related factors had only a limited influence on treatment outcomes. Normal body mass index and younger age were associated with greater improvement in selected outcome domains, whereas radiographic OA severity within moderate stages did not significantly affect treatment response. Cell dose, assessed via both total nucleated cell count and colony-forming unit indicators, showed significant associations with selected outcome domains, particularly Sport and Recreation and Quality of Life at 12 months. Importantly, no serious adverse events or procedure-related complications were observed, supporting the favorable safety profile of the intervention.

This study demonstrates an association between intra-articular autologous SVF therapy and significant improvements in pain, function, and quality of life in patients with moderate knee OA, with these effects largely maintained over a one-year follow-up. These findings suggest that adipose-derived stromal cell therapy may represent a safe complementary treatment option for patients with moderate knee osteoarthritis who seek alternatives to more invasive interventions. However, further prospective controlled studies with larger patient cohorts, longer follow-up periods, and standardized treatment and rehabilitation protocols are required to clarify long-term effectiveness, optimize dosing strategies, and define appropriate patient selection criteria.

## Figures and Tables

**Table 1:** Basic characteristics of the sample (sex, age, BMI, VAS and KOOS according to OA grade).

Variable/Category	All Patients	KL II	KL III
Number of participants [N (percentage)]	48 (100%)	16 (33.30%)	32 (66.70%)
Gender [male/female]	18/30	6/10	12/20
Age [mean ± SD]	53.88 ± 10.93	54.5 ± 10.46	53.56 ± 10.89
BMI [mean ± SD]	29.14 ± 4.58	27.46 ± 4.62	29.98 ± 4.64
Normal BMI (18.5–24.99) [n (percentage)]	4 (8.33%)	3 (18.80%)	1 (3.10%)
Elevated BMI (≥25) [n (percentage)]	44 (91.67%)	13 (81.30%)	31 (96.90%)
VAS (before treatment) [mean ± SD]	5.79 ± 1.69	5.25 ± 1.65	6.06 ± 1.66
KOOS (before treatment) [mean ± SD]	19.8 ± 9.22	19.97 ± 9.24	20.29 ± 9.04

Legend: N = number of participants; BMI = Body Mass Index; SD = standard deviation.

**Table 2:** Mean KOOSs, Friedman test results and effect sizes across time points.

KOOS Domain	0 m (N = 48) M ± SD /Mdn (IQR)	3 m (N = 48) M ± SD /Mdn (IQR)	12 m (N = 44) M ± SD /Mdn (IQR)	Friedman *p*	Post Hoc 0→3 m p_adj	Post Hoc 0→12 m p_adj	Post Hoc 3→12 m p_adj	d (0→3 m) [95% CI]	d (0→12 m) [95% CI]	d (3→12 m) [95% CI]
PAIN	24.48 ± 13.58 /23.6 (13.9–33.3)	63.83 ± 17.84 /65.3 (52.8–77.8)	73.30 ± 15.43 /75.0 (61.1–86.1)	<0.001	<0.001 ***	<0.001 ***	0.025 *	6.09 [4.89, 7.41]	2.65 [2.05, 3.32]	0.41 [0.14, 0.76]
SYMPTOMS	27.23 ± 17.49 /25.0 (10.7–39.3)	68.15 ± 20.99 /67.9 (53.6–85.7)	62.34 ± 23.63 /67.9 (46.4–82.1)	<0.001	<0.001 ***	<0.001 ***	0.888	6.72 [5.26, 7.97]	1.21 [0.86, 1.65]	−0.22 [−0.49, 0.11]
ADL	20.83 ± 12.82 /16.9 (11.8–29.4)	66.91 ± 15.27 /64.0 (55.9–79.4)	75.10 ± 14.73 /75.7 (67.6–86.8)	<0.001	<0.001 ***	<0.001 ***	0.066	10.18 [8.20, 12.35]	2.72 [2.09, 3.38]	0.34 [0.06, 0.67]
SPORT	14.06 ± 14.93 /10.0 (0.0–25.0)	56.35 ± 23.36 60.0 (40.0–75.0)	67.73 ± 29.38 /85.0 (45.0–90.0)	<0.001	<0.001 ***	<0.001 ***	0.032 *	3.61 [2.87, 4.43]	1.63 [1.26, 2.19]	0.31 [0.05, 0.65]
QoL	12.37 ± 14.90 /6.3 (0.0–18.8)	53.39 ± 22.33 /50.0 (37.5–68.8)	68.18 ± 34.68 /87.5 (37.5–93.8)	<0.001	<0.001 ***	<0.001 ***	0.024 *	3.59 [2.84, 4.40]	1.61 [1.19, 2.09]	0.42 [0.12, 0.74]

Legend: N = number of participants; M = mean; SD = standard deviation; Mdn = median; IQR = interquartile range (25th–75th percentile); d = paired Cohen’s d; CI = confidence interval. The Friedman test assessed the overall time effect (df = 2). Post hoc comparisons: Wilcoxon signed-rank tests with Bonferroni correction (adjusted α = 0.05/3 = 0.017). *** *p* < 0.001; * *p* < 0.05.

**Table 3:** Responder analysis—proportion of patients achieving MCID at 3 and 12 months.

KOOS Domain	MCID	3 m Resp.	3 m %	12 m Resp.	12 m %	12 m Worsened	12 m %W
Pain	12	47/48	97.9%	43/44	97.7%	0/44	0.0%
Symptoms	9	48/48	100%	35/44	79.5%	4/44	9.1%
ADL	10	48/48	100%	44/44	100%	0/44	0.0%
Sport/Rec	9	46/48	95.8%	41/44	93.2%	1/44	2.3%
QoL	14	46/48	95.8%	38/44	86.4%	3/44	6.8%

Legend: Resp. = responders (achieving the domain-specific MCID threshold); Worsened = patients with negative change; %W = percentage worsened. MCID = Minimal Clinically Important Difference.

**Table 4:** Spearman’s correlation coefficient assessing the association between participant OA grade (KL) and the change in KOOS domain scores across time points.

Correlation Between Variables	Domain Change 3–0 m (N = 48) r_s_ (*p*)	Domain Change 12–0 m (N = 44) r_s_ (*p*)	Domain Change 12–3 m (N = 44) r_s_ (*p*)
KL & PAIN	−0.070 (0.634)	0.000 (1)	0.075 (0.629)
KL & SYMPTOMS	0.019 (0.897)	0.086 (0.559)	−0.202 (0.188)
KL & SPORT	0.005 (0.974)	−0.011 (0.940)	−0.089 (0.565)
KL & ADL	0.011 (0.940)	−0.021 (0.889)	−0.029 (0.849)
KL & QoL	−0.235 (0.108)	−0.244 (0.095)	0.158 (0.304)

Legend: N—number of participants; KL—Kellgren–Lawrence OA grade; r_s_—Spearman rho; *p*—significance.

**Table 5:** Linear regression assessing the association between participant BMI and the change in KOOS domain scores across time points.

Association Between Variables	Domain Change 3–0 m (N = 48) t (*p*)	Domain Change 12–0 m (N = 44) t (*p*)	Domain Change 12–3 m (N = 44) t (*p*)
BMI & PAIN	0.690 (0.494)	1.649 (0.107)	1.279 (0.208)
BMI & SYMPTOMS	1.095 (0.279)	0.456 (0.651)	0.277 (0.783)
BMI & SPORT	0.942 (0.035 *)	1.275 (0.209)	0.862 (0.394)
BMI & ADL	−0.001 (0.999)	0.418 (0.678)	0.381 (0.705)
BMI & QoL	0.489 (0.627)	1.596 (0.118)	1.275 (0.209)

Legend: N—number of participants; BMI—Body Mass Index; *p*—significance. * *p* < 0.05.

**Table 6:** Spearman’s correlation coefficient assessing the association between participant age and the difference in KOOS domain scores across time points.

Correlation Between Variables	Domain Change 3–0 m (N = 48) r_s_ (*p*)	Domain Change 12–0 m (N = 44) r_s_ (*p*)	Domain Change 12–3 m (N = 44) r_s_ (*p*)
Age & PAIN	0.074 (0.616)	−0.053 (0.733)	−0.068 (0.663)
Age & SYMPTOMS	−0.029 (0.844)	0.091 (0.559)	0.092 (0.554)
Age & SPORT	−0.006 (0.969)	−0.277 (0.069)	−0.216 (0.159)
Age & ADL	−0.034 (0.816)	−0.138 (0.373)	−0.137 (0.376)
Age & QoL	−0.283 (0.063)	0.069 (0.643)	−0.335 (0.026 *)

Legend: N—number of participants; r_s_—Spearman rho; *p*—significance. * *p* < 0.05.

**Table 7:** Pearson’s *t*-test assessing the association between participant gender and the difference in KOOS domain scores across time points.

Association Between Variables	Domain Change 3–0 m (N = 48) t (*p*)	Domain Change 12–0 m (N = 44) t (*p*)	Domain Change 12–3 m (N = 44) t (*p*)
Gender & PAIN	0.875 (0.386)	0.762 (0.450)	0.386 (0.701)
Gender & SYMPTOMS	−0.237 (0.814)	1.533 (0.133)	1.432 (0.159)
Gender & SPORT	0.473 (0.638)	0.111 (0.913)	−0.031 (0.976)
Gender & ADL	−0.638 (0.526)	0.695 (0.491)	0.700 (0.488)
Gender & QoL	−0.940 (0.352)	−0.563 (0.576)	−0.341 (0.735)

Legend: N—number of participants; t—*t*-test statistics; *p*—significance.

**Table 8:** SVF product characteristics (N = 48) and correlation with patients’ BMI.

SVF Product Characteristics	Range	M ± SD	Correlation with BMI (Spearman’s Rho, *p*)
No. of nucleated cells (TNC)/mL of fat tissue (10^6^/mL)	0.09–1.75	0.61 ± 0.37	0.429, *p* = 0.002
No. of nucleated cells (TNC) in cell product (10^6^)	3.12–106.14	45.72 ± 27.05	0.369, *p* = 0.010
No. of stromal cells (CFU-F)/mL of fat tissue (10^6^/mL)	0.000015–0.05	0.005 ± 0.009	0.242, *p* = 0.098
No. of stromal cells (CFU-F) in cell product (10^6^)	0.0002–4.33	0.390 ± 0.753	0.250, *p* = 0.086
No. of stromal cells (CFU-F)/10^6^ nucleated cells	30.59–52,630.59	7458.532 ± 10,905.934	0.260, *p* = 0.074

Legend: N—number of participants; CFU-F—colony-forming unit–fibroblast; M—mean value; SD—standard deviation; range—minimum and maximum observed values.

**Table 9:** Spearman’s correlation coefficient for assessing the relationship between SVF dose data and KOOSs (PAIN, SYMPTOMS, SPORT, ADL, QoL) changes 3 months and 12 months after treatment.

Correlation Between Variables	KOOS Domain	Domain Change 3–0 m (N = 48) r_s_ (*p*)	Domain Change 12–0 m (N = 44) r_s_ (*p*)	Domain Change 12–3 m (N = 44) r_s_ (*p*)
No. of nucleated cells (TNC)/mL of fat tissue (10^6^/mL)	PAIN	0.129 (0.384)	0.074 (0.632)	0.041 (0.792)
	SYMPTOMS	−0.052 (0.725)	0.278 (0.068)	0.220 (0.152)
	SPORT	0.225 (0.125)	0.358 (0.017 *)	0.258 (0.090)
	ADL	−0.013 (0.932)	0.183 (0.233)	0.165 (0.284)
	QoL	0.198 (0.177)	0.277 (0.069)	0.221 (0.149)
No. of nucleated cells (TNC) in cell product (10^6^)	PAIN	0.120 (0.418)	0.087 (0.572)	0.061 (0.692)
	SYMPTOMS	−0.092 (0.533)	0.274 (0.072)	0.230 (0.133)
	SPORT	0.234 (0.109)	0.377 (0.012 *)	0.272 (0.075)
	ADL	0.020 (0.890)	0.147 (0.342)	0.124 (0.423)
	QoL	0.198 (0.176)	0.328 (0.030 *)	0.265 (0.082)
No. of stromal cells (CFU-F)/mL of fat tissue (10^6^/mL)	PAIN	−0.045 (0.762)	0.088 (0.569)	0.061 (0.695)
	SYMPTOMS	0.083 (0.577)	−0.002 (0.988)	−0.020 (0.898)
	SPORT	0.121 (0.413)	0.256 (0.094)	0.204 (0.185)
	ADL	0.035 (0.814)	0.096 (0.537)	0.062 (0.689)
	QoL	−0.003 (0.986)	0.449 (0.002 **)	0.394 (0.008 **)
No. of stromal cells (CFU-F) in cell product (10^6^)	PAIN	−0.043 (0.770)	0.076 (0.622)	0.055 (0.725)
	SYMPTOMS	0.074 (0.619)	0.001 (0.995)	−0.011 (0.942)
	SPORT	0.131 (0.377)	0.259 (0.089)	0.201 (0.190)
	ADL	0.043 (0.773)	0.085 (0.582)	0.053 (0.732)
	QoL	0.000 (1.000)	0.453 (0.002 **)	0.399 (0.007 **)
No. of stromal cells (CFU-F)/10^6^ nucleated cells	PAIN	−0.062 (0.677)	0.058 (0.708)	0.047 (0.763)
	SYMPTOMS	0.098 (0.507)	−0.106 (0.494)	−0.105 (0.497)
	SPORT	0.051 (0.731)	0.143 (0.356)	0.119 (0.442)
	ADL	0.086 (0.563)	−0.008 (0.957)	−0.032 (0.834)
	QoL	−0.113 (0.446)	0.409 (0.006 **)	0.375 (0.012 *)

Legend: N—number of participants; r_s_—Spearman’s rank correlation coefficient (rho); *p*—significance. ** *p* < 0.01; * *p* < 0.05.

**Table 10:** Multiple linear regression—predictors of KOOS change at 12 months (adjusted for baseline).

Domain	Predictor	B	SE	β	t	*p*	Adj. R^2^
Pain	Baseline	−0.787	0.187	−0.584	−4.22	<0.001 **	0.240
	Other pred.	—	—	—	—	all ns	
Symptoms	BMI	−2.070	0.835	−0.341	−2.48	0.018 *	0.367
	Baseline	−0.938	0.213	−0.549	−4.41	<0.001 **	
ADL	Baseline	−1.015	0.179	−0.686	−5.66	<0.001 **	0.392
	Other pred.	—	—	—	—	all ns	
Sport/Rec	Baseline	−0.769	0.317	−0.365	−2.43	0.020 *	0.100
	Other pred.	—	—	—	—	all ns	
QoL	Age	−0.990	0.464	−0.320	−2.13	0.040 *	0.134
	Cell dose	16.887	6.973	0.361	2.42	0.021 *	

Legend: B = unstandardized coefficient; SE = standard error; β = standardized coefficient; only significant predictors shown per domain. All models adjusted for age, sex, BMI, KL grade, cell dose (CFU-F), and baseline KOOS. ** *p* < 0.01; * *p* < 0.05.

## Data Availability

The original data presented in the study are openly available in repository ReVIS: http://hdl.handle.net/20.500.12556/ReVIS-13270 (accessed on 9 March 2026).

## Abbreviations

The following abbreviations are used in this manuscript:

ADL	Activities of Daily Living
AD-MSC	adipose-derived stromal cells
BMI	Body Mass Index
CFU-F	colony-forming unit–fibroblast
CT	computed tomography
FAOS	Foot and Ankle Outcome Score
GMP-grade	Good Manufacturing Practice
IL-6	Interleukin-6
KL	Kellgren–Lawrence (grading system)
KOOS	Knee Injury and Osteoarthritis Outcome Score
M	mean value
MCP-1	Monocyte Chemoattractant Protein-1
MRI	magnetic resonance imaging
MSC	mesenchymal stromal cells
N	number of participants
OA	osteoarthritis
QoL	Quality of Life
Rs	Spearman’s rank correlation coefficient
SD	standard deviation
SPSS	Statistical Package for the Social Sciences (SPSS software)
SVF	stromal vascular fraction
TNC	total nucleated cell count
VAS	Visual Analog Scale

## References

[1] Henning M. (2022). Definition of Early Osteoarthritis. Early Osteoarthritis: State-of-the-Art Approaches to Diagnosis, Treatment and Controversies.

[2] Mijatović I., Moličnik A. (2021). Artroza Velikih Sklepov: Epidemiologija, Etiologija in Patofiziologija.

[3] Piñeiro-Ramil M., Castro-Viñuelas R., Sanjurjo-Rodríguez C., Hermida-Gómez T., Fuentes-Boquete I., de Toro-Santos F.J., Blanco-García F.J., Díaz-Prado S.M., Zorzi A.R., Batista de Miranda J. (2018). Cell Therapy and Tissue Engineering for Cartilage Repair. Cartilage Repair and Regeneration.

[4] Steenkamp W., Rachuene P.A., Dey R., Mzayiya N.L., Ramasuvha B.E. (2022). The Correlation between Clinical and Radiological Severity of Osteoarthritis of the Knee. SICOT-J.

[5] Giorgino R., Albano D., Fusco S., Peretti G.M., Mangiavini L., Messina C. (2023). Knee Osteoarthritis: Epidemiology, Pathogenesis, and Mesenchymal Stem Cells: What Else Is New? An Update. Int. J. Mol. Sci..

[6] Innmann M.M., Lunz A., Fröhlich L., Bruckner T., Merle C., Reiner T., Schiltenwolf M. (2023). What Is the Correlation between Clinical and Radiographic Findings in Patients with Advanced Osteoarthritis of the Knee?. J. Clin. Med..

[7] Arendt E.A., Parker D. (2016). Osteoarthritis: Definition, Etiology, and Natural History. Management of Knee Osteoarthritis in the Younger, Active Patient: An Evidence-Based Practical Guide for Clinicians.

[8] Carneiro D.D.C., de Araújo L.T., Santos G.C., Damasceno P.K.F., Vieira J.L., dos Santos R.R., Barbosa J.D.V., Soares M.B.P. (2023). Clinical Trials with Mesenchymal Stem Cell Therapies for Osteoarthritis: Challenges in the Regeneration of Articular Cartilage. Int. J. Mol. Sci..

[9] Butler D.S., Moseley G.L. (2015). Explain Pain.

[10] Rodríguez-Merchán E.C. (2022). Intraarticular Injections of Mesenchymal Stem Cells in Knee Osteoarthritis: A Review of Their Current Molecular Mechanisms of Action and Their Efficacy. Int. J. Mol. Sci..

[11] He W., Kuang M., Zhao J., Sun L., Lu B., Wang Y., Ma J., Ma X. (2017). Efficacy and Safety of Intraarticular Hyaluronic Acid and Corticosteroid for Knee Osteoarthritis: A Meta-Analysis. Int. J. Surg..

[12] Hussein M., Van Eck C.F., Kregar Velikonja N. (2021). Bone Marrow Aspirate Concentrate Is More Effective Than Hyaluronic Acid and Autologous Conditioned Serum in the Treatment of Knee Osteoarthritis: A Retrospective Study of 505 Consecutive Patients. Appl. Sci..

[13] Delco M.L., Srivastava N., Lattermann C., Henning M., Norimasa N., Kon E. (2021). Mesenchymal Stromal Cells and Extracellular Vesicles. Early Osteoarthritis: State-of-the-Art Approaches to Diagnosis, Treatment and Controversies.

[14] Parker D.A., Scholes C., Parker D.A. (2016). Nonoperative Treatment Options for Knee Osteoarthritis. Management of Knee Osteoarthritis in the Younger, Active Patient: An Evidence-Based Practical Guide for Clinicians.

[15] McKay J., Frantzen K., Vercruyssen N., Hafsi K., Opitz T., Davis A., Murrell W. (2019). Rehabilitation Following Regenerative Medicine Treatment for Knee Osteoarthritis-Current Concept Review. J. Clin. Orthop. Trauma.

[16] Centeno C.J., Pastoriza S.M. (2020). Past, current and future interventional orthobiologics techniques and how they relate to regenerative rehabilitation: A clinical commentary. Int. J. Sports Phys. Ther..

[17] Primorac D., Molnar V., Rod E., Jeleč Ž., Čukelj F., Matišić V., Vrdoljak T., Hudetz D., Hajsok H., Borić I. (2020). Knee Osteoarthritis: A Review of Pathogenesis and State-Of-The-Art Non-Operative Therapeutic Considerations. Genes.

[18] Czerwiec K., Zawrzykraj M., Deptuła M., Skoniecka A., Tymińska A., Zieliński J., Kosiński A., Pikuła M. (2023). Adipose-Derived Mesenchymal Stromal Cells in Basic Research and Clinical Applications. Int. J. Mol. Sci..

[19] Wei P., Bao R. (2022). Intra-Articular Mesenchymal Stem Cell Injection for Knee Osteoarthritis: Mechanisms and Clinical Evidence. Int. J. Mol. Sci..

[20] Chen Y., Cheng R.-J., Wu Y., Huang D., Li Y., Liu Y. (2023). Advances in Stem Cell-Based Therapies in the Treatment of Osteoarthritis. Int. J. Mol. Sci..

[21] Zupan J., Čamernik K., Jeras M., Barlič A., Drobnič M., Marc J. (2017). Mezenhimske Matične Celice: Uporaba in Potencial za Zdravljenje ter Diagnostiko Mišično-Skeletnih Bolezni. Farm. Vestn..

[22] Maldonado V.V., Patel N.H., Smith E.E., Barnes C.L., Gustafson M.P., Rao R.R., Samsonraj R.M. (2023). Clinical Utility of Mesenchymal Stem/Stromal Cells in Regenerative Medicine and Cellular Therapy. J. Biol. Eng..

[23] Xiang X.-N., Zhu S.-Y., He H.-C., Yu X., Xu Y., He C.-Q. (2022). Mesenchymal Stromal Cell-Based Therapy for Cartilage Regeneration in Knee Osteoarthritis. Stem Cell Res. Ther..

[24] Li C., Zhao H., Cheng L., Wang B. (2021). Allogeneic vs. Autologous Mesenchymal Stem/Stromal Cells in Their Medication Practice. Cell Biosci..

[25] Yin P., Jiang Y., Fang X., Wang D., Li Y., Chen M., Deng H., Tang P., Zhang L. (2023). Cell-Based Therapies for Degenerative Musculoskeletal Diseases. Adv. Sci..

[26] Zhuang W.-Z., Lin Y.-H., Su L.-J., Wu M.-S., Jeng H.-Y., Chang H.-C., Huang Y.-H., Ling T.-Y. (2021). Mesenchymal Stem/Stromal Cell-Based Therapy: Mechanism, Systemic Safety and Biodistribution for Precision Clinical Applications. J. Biomed. Sci..

[27] Lv Z., Cai X., Bian Y., Wei Z., Zhu W., Zhao X., Weng X. (2023). Advances in Mesenchymal Stem Cell Therapy for Osteoarthritis: From Preclinical and Clinical Perspectives. Bioengineering.

[28] Jeyaraman M., Maffulli N., Gupta A. (2023). Stromal Vascular Fraction in Osteoarthritis of the Knee. Biomedicines.

[29] Huang Z., Zhang S., Cao M., Lin Z., Kong L., Wu X., Guo Q., Ouyang Y., Song Y. (2023). What Is the Optimal Dose of Adipose-Derived Mesenchymal Stem Cells Treatment for Knee Osteoarthritis? A Conventional and Network Meta-Analysis of Randomized Controlled Trials. Stem Cell Res. Ther..

[30] Alonso-Goulart V., Carvalho L.N., Marinho A.L.G., De Oliveira Souza B.L., De Aquino Pinto Palis G., Lage H.G.D., De Lima I.L., Guimarães L.D., Peres L.C., Silveira M.M. (2021). Biomaterials and Adipose-Derived Mesenchymal Stem Cells for Regenerative Medicine: A Systematic Review. Materials.

[31] Goncharov E.N., Koval O.A., Nikolaevich Bezuglov E., Encarnacion Ramirez M.D.J., Engelgard M., Igorevich E.I., Saporiti A., Valentinovich Kotenko K., Montemurro N. (2023). Stromal Vascular Fraction Therapy for Knee Osteoarthritis: A Systematic Review. Medicina.

[32] Gao S., Zhao K., Lu Z. (2025). A Review of Clinical Studies of Stromal Vascular Fraction for the Treatment of Osteoarthritis with a Follow-up Period of Over Two Years. Stem Cell Rev. Rep..

[33] Ferreira M.Y., Carvalho J.D.C., Ferreira L.M. (2023). Evaluating the Quality of Studies Reporting on Clinical Applications of Stromal Vascular Fraction: A Systematic Review and Proposed Reporting Guidelines (CLINIC-STRA-SVF). Regen. Ther..

[34] Roos E.M., Lohmander L.S. (2003). The Knee Injury and Osteoarthritis Outcome Score (KOOS): From Joint Injury to Osteoarthritis. Health Qual. Life Outcomes.

[35] Boffa A., Andriolo L., Franceschini M., Di Martino A., Asunis E., Grassi A., Zaffagnini S., Filardo G. (2021). Minimal Clinically Important Difference and Patient Acceptable Symptom State in Patients with Knee Osteoarthritis Treated with PRP Injection. Orthop. J. Sports Med..

[36] Migliorini F., Maffulli N., Schäfer L., Simeone F., Bell A., Hofmann U.K. (2024). Minimal Clinically Important Difference (MCID), Substantial Clinical Benefit (SCB), and Patient-Acceptable Symptom State (PASS) in Patients Who Have Undergone Total Knee Arthroplasty: A Systematic Review. Knee Surg. Relat. Res..

[37] Iijima H., Isho T., Kuroki H., Takahashi M., Aoyama T. (2018). Effectiveness of Mesenchymal Stem Cells for Treating Patients with Knee Osteoarthritis: A Meta-Analysis toward the Establishment of Effective Regenerative Rehabilitation. npj Regen. Med..

[38] Mautner K., Bowers R., Easley K., Fausel Z., Robinson R. (2019). Functional Outcomes Following Microfragmented Adipose Tissue Versus Bone Marrow Aspirate Concentrate Injections for Symptomatic Knee Osteoarthritis. Stem Cells Transl. Med..

[39] Conley S.M., Hickson L.J., Kellogg T.A., McKenzie T., Heimbach J.K., Taner T., Tang H., Jordan K.L., Saadiq I.M., Woollard J.R. (2020). Human Obesity Induces Dysfunction and Early Senescence in Adipose Tissue-Derived Mesenchymal Stromal/Stem Cells. Front. Cell Dev. Biol..

[40] Yang H.J., Kim K.-J., Kim M.K., Lee S.J., Ryu Y.H., Seo B.F., Oh D.-Y., Ahn S.-T., Lee H.Y., Rhie J.W. (2014). The Stem Cell Potential and Multipotency of Human Adipose Tissue-Derived Stem Cells Vary by Cell Donor and Are Different from Those of Other Types of Stem Cells. Cells Tissues Organs.

[41] Jeyaraman M., Muthu S., Nischith D.S., Jeyaraman N., Nallakumarasamy A., Khanna M. (2022). PRISMA-Compliant Meta-Analysis of Randomized Controlled Trials on Osteoarthritis of Knee Managed with Allogeneic vs Autologous MSCs: Efficacy and Safety Analysis. Indian J. Orthop..

[42] Jeyaraman M., Muthu S., Ganie P.A. (2021). Does the Source of Mesenchymal Stem Cell Have an Effect in the Management of Osteoarthritis of the Knee? Meta-Analysis of Randomized Controlled Trials. Cartilage.

[43] Qu H., Sun S. (2021). Efficacy of Mesenchymal Stromal Cells for the Treatment of Knee Osteoarthritis: A Meta-Analysis of Randomized Controlled Trials. J. Orthop. Surg. Res..

[44] Kolar M., Veber M., Girandon L., Drobnič M. (2024). Biomaterials Augmented with Filtered Bone Marrow Aspirate for the Treatment of Talar Osteochondral Lesions. A Comparison of Clinical and Cellular Parameters. J. Orthop. Surg..

[45] Vogt A., Faher A., Kucharczak J., Birch M., McCaskie A., Khan W. (2024). The Effects of Gender on Mesenchymal Stromal Cell (MSC) Proliferation and Differentiation In Vitro: A Systematic Review. Int. J. Mol. Sci..

[46] Kim K.-I., Kim M.-S., Kim J.-H. (2023). Intra-Articular Injection of Autologous Adipose-Derived Stem Cells or Stromal Vascular Fractions: Are They Effective for Patients with Knee Osteoarthritis? A Systematic Review with Meta-Analysis of Randomized Controlled Trials. Am. J. Sports Med..

[47] Rogers C.J., Harman R., Sheinkop M.B., Hanson P., Ambach M.A., David T., Desai R., Sampson S., Aufierro D., Bowen J. (2024). Clinical Evaluation of Safety and Efficacy of a Central Current Good Manufacturing Practices Laboratory Produced Autologous Adipose-Derived Stromal Vascular Fraction Cell Therapy Product for the Treatment of Knee Osteoarthritis. Stem Cells Dev..

[48] Totlis T., Achlatis V., Emfietzis P., Marín Fermín T., Pettas T., Sideridis A., Terzidis I. (2025). A Single Intra-articular Stromal Vascular Fraction with Platelet-rich Plasma Injection Yields Superior Clinical Outcomes than a Hyaluronic Acid Injection in Patients with Knee Osteoarthritis: A Prospective Comparative Study. Knee Surg. Sports Traumatol. Arthrosc..

[49] Mangiavini L., Rossi N., Giorgino R., Borelli F., Colombini A., De Luca P., Landoni S., Sconfienza L.M., Messina C., Beriotto I. (2025). Autologous Minimally Manipulated Adipose-derived Stromal Vascular Fraction in Knee Osteoarthritis: Lasting Symptom Relief and Imaging Evidence from a 12-month Prospective Study. Knee Surg. Sports Traumatol. Arthrosc..

[50] Green S.B. (1991). How Many Subjects Does it Take to Do a Regression Analysis. Multivar. Behav. Res..

